# New Insights Into Human Hyaluronidase 4/Chondroitin Sulphate Hydrolase

**DOI:** 10.3389/fcell.2021.767924

**Published:** 2021-10-20

**Authors:** Marissa L. Maciej-Hulme

**Affiliations:** Department of Nephrology, Radboud University Medical Center, Nijmegen, Netherlands

**Keywords:** chondroitin sulphate, proteoglycan, hydrolase, cancer, stem cell, spinal cord injury, osteoarthritis, catabolism

## Abstract

In this review, the current experimental evidence, literature and hypotheses surrounding hyaluronidase 4 [HYAL4, also known as chondroitin sulphate hydrolase (CHSE)] and chondroitin sulphate (CS) are explored. Originally named for its sequence similarity to other members of the hyaluronidase family, HYAL4 is actually a relatively distinct member of the family, particularly for its unique degradation of CS-D (2-*O*-, 6-*O*-sulphated CS) motifs and specific expression. Human HYAL4 protein expression and structural features are discussed in relation to different isoforms, activities, potential localisations and protein-protein interaction partners. CS proteoglycan targets of HYAL4 activity include: serglycin, aggrecan, CD44 and sulfatase 2, with other potential proteoglycans yet to be identified. Importantly, changes in HYAL4 expression changes in human disease have been described for testicular, bladder and kidney cancers, with gene mutations reported for several others including: leukaemia, endometrial, ovarian, colorectal, head and neck, stomach, lung and breast cancers. The HYAL4 gene also plays a role in P53 negative human cancer cell proliferation and is linked to stem cell naivety. However, its role in cancer remains relatively unexplored. Finally, current tools and techniques for the detection of specific HYAL4 activity in biological samples are critically assessed. Understanding the role of HYAL4 in human diseases will fortify our understanding of developmental processes and disease manifestation, ultimately providing novel diagnostic opportunities and therapeutic targets for drug discovery.

## Introduction

### Structure of Chondroitin Sulphate/Dermatan Sulphate (CSPGs)

The glycosaminoglycan, chondroitin sulphate (CS), is a long, linear polysaccharide comprised of repeating glucuronic acid-*N*-acetylgalactosamine (GlcA-GalNAc) disaccharides that adorn a subset of glycosylated proteins, termed CS proteoglycans (CSPGs). Members of the family play diverse roles in tissue architecture, cell signalling, cell migration and growth, as well as in disease manifestation including: inflammation, cancer, neurological diseases and osteoarthritis (Bautch et al., 2000; Kim et al., 2011; Chi et al., 2012; Mikami and Kitagawa, 2013; Mizumoto et al., 2015; Hayes et al., 2016; Stephenson and Yong, 2018; Shida et al., 2019; Lokeshwar et al., 2020). The CSPG family has recently expanded to include 19 newly identified CS attachment sites in the human proteome (Noborn et al., 2021). CS chains are built on to a common tetrasaccharide linker (xylose-galactose-galactose-GlcA) in the endoplasmic reticulum and Golgi apparatus, and are tethered to the protein core via a serine residue. A myriad of enzymes construct CS chains [reviewed extensively in Mikami and Kitagawa (2013)] and multiple sulphate groups may be positioned along the CS polymer, namely 2-*O*-sulphation on the GlcA, and/or 4-*O*- and 6-*O*-sulphation on the GalNAc, respectively. These sulphation modifications give rise to great structural diversity, forming functional motifs that participate in CS-ligand interactions. In addition, epimerisation of GlcA to iduronic acid within the chain creates hybrid CS/dermatan sulphate (DS) chains, altering the flexibility of the polymer and consequentially potential ligand interactions (Thelin et al., 2013; Mizumoto et al., 2015). Classification of unsulphated chondroitin, CS and DS chains is defined by the chemical structure of the polymer, with CS split into subtypes: CS-A (4-*O*-sulphated), CS-C (6-*O*-sulphated), CS-D (2-*O*-, 6-*O*-sulphated), and CS-E (4-*O*-, 6-*O*-sulphated). The latter two subtypes represent rarer sulphation modifications present within CS/DS chains. CS bioactivity is often described for CS/DS hybrid chains, and CS enriched in rarer sulphation types, such as CS-D and CS-E (Bao et al., 2004; Kim et al., 2011; Beurdeley et al., 2012; Mizumoto et al., 2015; Shida et al., 2019).

**FIGURE 1:**
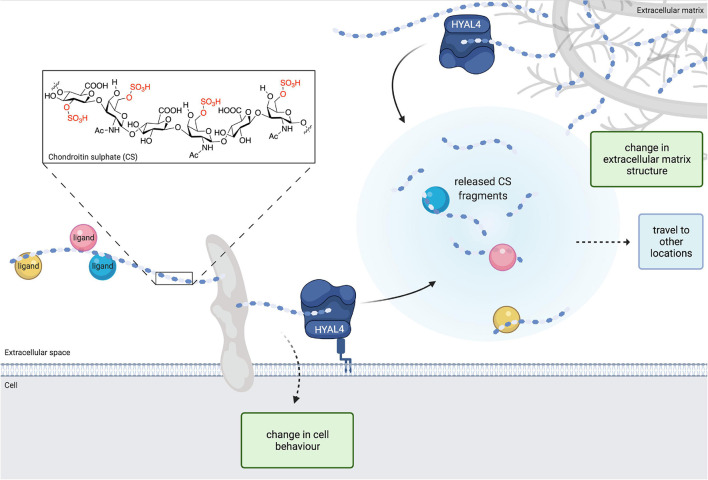
The structure of chondroitin sulphate (CS) is modified by hyaluronidase 4 (HYAL4)/chondroitin sulphate hydrolase (CHSE) degradation activity. Image depicts HYAL4 modification of cell surface and extracellular matrix CS proteoglycans (CSPGs). CS side chains are cleaved by HYAL4 to produce free oligosaccharides and a shorter parent chain. Released CS fragments and ligand cargo are free to travel to different locations and exert changes on other cells. The modified parent CSPG also modifies cell behaviour. Removal of CS from the extracellular matrix induces changes in tissue structure. Inset, chemical structure of CS with example sulphate groups highlighted in red. Figure created with BioRender.com.

After biosynthesis is complete, further modification of CS chains can occur via degradation enzymes that cleave the glycosidic bonds between the saccharide units and release CS fragments from the parent polymer. Three human extra-lysosomal CS hydrolases have been identified: PH20 (SPAM1), hyaluronidase 1 (HYAL1) and hyaluronidase 4 [HYAL4, also known as chondroitin sulphate hydrolase (CHSE)] (Kaneiwa et al., 2010; Honda et al., 2012; Yamada, 2015). Unlike the first two that can degrade hyaluronic acid and CS substrates to a similar degree (Honda et al., 2012), HYAL4 is predominantly an endo-β-*N*-acetylgalactosaminidase with a strong preference for CS-D (2-*O*-, 6-*O*-sulphated CS) (Kaneiwa et al., 2010; Wu and Ertelt, 2021). Although HYAL4 CHSE activity was only discovered in 2010, a few CSPGs have already been identified to be modified by HYAL4 degradation including serglycin, aggrecan (Farrugia et al., 2019), CD-44 (Lokeshwar et al., 2020) and likely Sulfatase 2 too (El Masri et al., 2021). HYAL4 cleavage of CSPGs produces smaller [tetra- to dodecasaccharide (Bautch et al., 2000; Bao et al., 2004; Kim et al., 2011; Mikami and Kitagawa, 2013; Thelin et al., 2013; Mizumoto et al., 2015; Hayes et al., 2016; Shida et al., 2019; Noborn et al., 2021) sized] fragments (Farrugia et al., 2019) with a common structure [GlcA-GalNAc(6S)-GlcA, also known as 3B3- motifs] located proximally at the non-reducing end of the chain (Figure 1).

## Human Hyaluronidase 4 Protein Expression and Structure

The HYAL4 gene is located on chromosome locus 7q31.3. Several tissues and cell types have been identified to express HYAL4, particularly placenta, skeletal muscle tissue and neutrophils (granulocytes) (Farrugia et al., 2019; Quirós et al., 2019). Northern blot analysis showed that expression of HYAL4 exists as 2.4- and 4.0-kb transcripts in placenta as well as a 2.1-kb transcript in skeletal muscle, suggesting differential splicing of the gene may occur in different cell types and/or contexts (Csoka et al., 1999). HYAL4 protein expression has also been identified for several tissues of which testes and skeletal muscle are enriched (Bastow et al., 2008; Elbein et al., 2011; Quirós et al., 2019). Immunohistochemical staining of human tissues showed HYAL4 in the cytoplasm and membrane of cells and indicated the presence of HYAL4 in neuronal cell nuclei (Uhlen et al., 2015). Flow cytometry of non-permeabilised mast cells showed HYAL4 to be associated with the cell surface (Farrugia et al., 2019). *In vitro* experiments have demonstrated that at least one protein isoform is released into the extracellular milieu (Lokeshwar et al., 2020). However, the habitual location(s) of HYAL4 remains to be established.

Four proteins have been predicted from the human gene, two of which have been confirmed experimentally. The full-length variant (481 amino acids, UniProt Q2M3T9) is currently predicted to be a transmembrane protein, with cytoplasmic N- and C-termini and an extracellular catalytic domain followed by a peptide linker. However, unlike HYAL1, HYAL2, and HYAL3, no obvious canonical signal sequence has been identified for targeting to the membrane. Furthermore, a putative GPI-anchor at the C-terminus (position 455) is predicted (Kaneiwa et al., 2010, 2012), which would subsequently remove the C-terminal transmembrane domain. In several ape species, pig and mouse, HYAL4 orthologs also have predicted GPI-anchors but in rats and *Caenorhabditis elegans* the protein is probably secreted. Interestingly, human HYAL2, and PH20 also have consensus GPI anchor sites, but HYAL1 and HYAL3 do not. The shorter 349 amino acid human v1 variant of HYAL4 (UniProt, F8WDH9) is a truncated version, omitting the C-terminal peptide linker, predicted transmembrane and cytoplasmic peptide domains, which suggests that this protein form is secreted.

Both identified HYAL4 variants possess catalytic activity. For the full-length version, CS-D motifs are the preferred substrate with an optimum pH of 4.5–5 (Kaneiwa et al., 2010). In contrast, the v1 variant preferred cleaving CS-C over CS-D and enzyme activity had an optimum pH of 5–5.5 (Lokeshwar et al., 2020), suggesting that some catalytic specificity of the substrate preference is encoded within the peptide linker, even though the mutation was not directly adjacent to the catalytic residue (E147). A single mutation in the positioning residue Y247 in human HYAL4 (equivalent to Y219 in TsHyal-1) results in altered substrate specificity of full length HYAL4 (Jedrzejas and Stern, 2005). The differences in CS catalytic specificities in the splice variants may act as an additional layer of regulation to CS biology in tandem with enzyme localisation (membrane bound/anchored vs. secretion) for diverse control of multiple CSPG targets. Other methods of catalytic control may lie in the post translational modifications of the protein. Phosphorylation and acetylation mechanisms for enzyme activation or deactivation have been described for many enzymes (Guan and Xiong, 2011; Ardito et al., 2017). Three potential phosphorylation sites (Y43, T88, and Y296) and one acetylation site (E193) close to the catalytic residue (E147) have been predicted, all located within the catalytic domain. The protein has four potential *N*-glycosylation sites (86, 115, 177, and 343 amino acid positions), with the presence of a complex *N*-glycan confirmed at 177 (Jia et al., 2009). Curiously, the peptide linker (and C-terminus) is what sets HYAL4 apart from other HYALs, and is a conserved feature in other species, including mice and the single CSHY present in *C. elegans* (Kaneiwa et al., 2008). This raises questions about its purpose for HYAL4 function via non-enzymatic mechanisms that have been described for other enzymes such as transportation of molecules, regulation and structural support (Kung and Jura, 2016).

In short, there are many unanswered questions surrounding HYAL4 protein structure. Whilst the structural features of the protein(s) remain unsolved, so do their functions. Hence the deduction of HYAL4 protein structure and the characterisation of post translational modifications of the protein will be an important corner stone for future studies in HYAL4 and CS/DS biology.

### Hyaluronidase 4 Protein-Protein Interactions

Interestingly, three distinct proteins have been identified as interaction partners of HYAL4: Glyceraldehyde-3-phosphate dehydrogenase, spermatogenic (GAPDHS; Huttlin et al., 2017), Isoleucine tRNA Synthetase 2 (IARS2; Wan et al., 2015) and NIMA Related Kinase 4 (NEK4; Basei et al., 2015). GAPDHS (also known as GAPDH-2) is an enzyme belonging to the Glyceraldehyde-3-phosphate dehydrogenase family that generates 1,3-diphosphoglycerate from glyceraldehyde-3-phosphate, and is thought to act as a switch between pathways for energy production. It is highly expressed in elongated (late) spermatids but has also been detected in malignant melanoma (Hoek et al., 2008), suggesting that its role is not confined to spermiogenesis as implied by its name. IARS2 is a ubiquitously expressed mitochondrial tRNA synthase that catalyses the aminoacylation of tRNA with isoleucine. Knockdown of IARS2 has been shown to promote apoptosis and inhibit proliferation in melanoma cells (Ma et al., 2020). NEK4 is a serine/threonine kinase involved in replicative senescence and for normal cell cycle arrest in response to double-stranded DNA damage (Nguyen et al., 2012). There are two splice variants of NEK4 (NEK4.1 and NEK4.2) (Basei et al., 2015) both with a nuclear localisation sequence in the regulatory domain (Hayashi et al., 1999) but cell cytoplasmic expression has also been observed. Expression of NEK4 is particularly abundant in Leydig cells of the testes as well as exocrine glandular cells of the pancreas, adrenal glandular cells of the stomach and adrenal gland. GAPDHS, IARS2, and NEK4 have no known links between each other and none have been previously associated with CS. Thus, until further experimental evidence is reported, their roles in HYAL4 (and CS) biology remain intriguing but speculative.

## Assays and Tools for the Detection of Hyaluronidase 4 Activity and Function

### Degradation Activity Assays

Since the discovery of HYAL4 activity on CS, several new antibodies and quantitation kits became commercially available for the protein, which will greatly support future HYAL4 research. However, detection and quantification of HYAL4 enzyme activity remains challenging, as it is not always straightforward to delineate specific enzyme CHSE activity from biological samples. Although PH20 expression appears to be largely restricted to testes, HYAL1 is more widely expressed and possesses CHSE activity for CS-A. Unfortunately, natural sources of CS are typically a mixture of sulphation types classified on their predominant species i.e., CS-A is predominantly 4-sulphated but also contains a small amount of other rarer CS unit types, such as CS-D. Furthermore, CS-D preparations are usually a type of CS-A enriched with CS-D units (e.g., 16% CS-D units from shark cartilage produced by Iduron, United Kingdom). So for determining HYAL1 CHSE activity, one could simply use CS-A as an optimal substrate with relative ease. But for HYAL4, the CS-D preparations have cleavable sites for both HYAL4 and HYAL1, meaning that both HYALs could degrade significant portions of CS-D and produce a positive result in the assay. To untangle this, (1) a parallel assay must be performed with CS-A to deduce whether HYAL1 CHSE activity is present in the sample, (2) careful consideration of the detection strategy (biotinylation, antibodies) is important to maximise detection of removed CS and therefore detection of desired activity, (3) in the case that HYAL1 CHSE activity is detected, other evidence (e.g., qPCR for gene expression, western blot, protein quantification ELISA) is necessary to verify the enzymes responsible. For situations where both HYAL1 and HYAL4 activity is detected, quantification of HYAL4 activity will not be possible until assays with specific substrates exclusive for HYAL4 activity can be developed. Therefore, new chemically synthesised substrates designed specifically for HYAL4 activity and tailored antibodies for CS-D units are much-needed tools to facilitate the measurement of HYAL4 activity in biological samples.

### Antibodies

The monoclonal antibody (mab) 3B3 was originally created to recognise the neoepitope of CS chains following bacterial lyase (cABCase) digestion (designated 3B3+, i.e., +cABCase digestion) (Couchman et al., 1984). However, native 3B3 (i.e., without cABCase digestion) motifs were also detected in subpopulations of proteoglycans in chick embryos (Sorrell et al., 1988), subsequently termed 3B3-. Indeed, other CSPG 3B3- motifs have been studied including: serglycin, aggrecan (Farrugia et al., 2019, 2020), and CSPGs in the synovial fluid of elderly osteoarthritic patients (Bautch et al., 2000), where a significant decline in 3B3 and CS is associated with aging and articular cartilage progenitor CSPG(s) (Hayes et al., 2008). In addition to 3B3, MO-225 specifically recognises 2-*O*-sulphation in CS-D (Ito et al., 2005) and thus may be a useful antibody for the identification of HYAL4 modulated CSPGs.

## Hyaluronidase 4 in Human Development and Disease

### Stem Cells and Differentiation

As mentioned earlier, the expression of human HYAL4 is somewhat limited in adult tissues. Beguilingly though, HYAL4 expression has been associated with stem cell naivety in human embryonic stem cells, suggesting a fundamental role in the delicate balance of cell cycle regulation, pluripotency and priming for differentiation (Messmer et al., 2019). 3B3- motifs decorate stem cell/progenitor cell proteoglycans (Hayes et al., 2018) and are located in discrete zones of foetal human knee joint during bone and cartilage development (Hayes et al., 2016). Specifically, HYAL4 expression increases during bone mineralisation, along with an increase in CS/DS chains and sulphation content of the chains (Adams et al., 2006). Expression of HYAL4 is also located distinctively at the epidermal-dermal junction in the skin (Sorrell et al., 1990), as well as being expressed following rat spinal cord injury and in a sheep intervertebral disc regeneration model, indicating its involvement in CSPG remodelling for tissue development and regeneration (Tachi et al., 2015; Farrugia et al., 2020). When taken in tandem with its limited constitutive adult tissue expression, the association of HYAL4 expression with naïve human embryonic stem cells and its temporal and/or localised expression in tissues, implies that HYAL4 activity may play transient, but important roles during organismal development.

### Cancer

In the Cancer Genome Atlas project database, HYAL4 gene mutations were associated with 12 out of the 15 cancer types analysed. In particular, roughly 4% of endometrial tumours had some kind of mutation in the HYAL4 gene that resulted in a disruption of protein structure. Other cancers with HYAL4 gene mutations included: colorectal, stomach, lung (adenocarcinoma, squamous), bladder, glioblastoma, leukaemia, head/neck, ovarian, breast and kidney (clear cell) cancers (∼0.25–1.8% prevalence) (Chi et al., 2012; Li et al., 2013; Lokeshwar et al., 2020; Hasanali et al., 2021). In addition, upregulation of HYAL4 protein was observed in testicular cancer (Lokeshwar et al., 2020) and the HYAL4 gene was preferentially required for the proliferation of P53 negative human cancer cells (Xie et al., 2012). On a protein level, interaction of HYAL4 with NEK4 may play an important part in the epithelial-to-mesenchymal transition of cells during the development of cancer. NEK4 is present in most primary carcinomas where it acts as a positive regulator for EMT, resulting in an increased potential for cancer cell migration and invasion (Ding et al., 2018). So far, only two of the HYAL4 mutated cancer types identified have been investigated in more detail. In kidney cancer tissue, HYAL4 mRNA expression was significantly increased in clear cell renal cell carcinomas, papillary tumours and chromophobe renal cell carcinomas when compared with oncocytomas and HYAL4 upregulation was increased in patients with metastasis (Chi et al., 2012). In bladder cancer, HYAL4 activity increased the release of CD44, MMP-9 and Akt signalling and corresponded with metastasis and/or death of the patient after follow up. The v1 protein variant also showed chemotherapeutic resistance to Gemcitabine in preclinical models, suggesting HYAL4 drives chemoresistance in bladder cancer (Lokeshwar et al., 2020; Hasanali et al., 2021). Transfection of the v1 isoform in normal bladder cells resulted in an increase in aldehyde dehydrogenase-1, cell motility in wound healing assays and upregulation of EMT invasive phenotype markers that are hallmarks of cancer stem cells, invasiveness and EMT, respectively (Lokeshwar et al., 2020). Notably, the commonality of HYAL4 shared between cancer proliferation and stem cell naivety points toward a function for HYAL4 in the development and/or maintenance of cancer stem cells, which often cause therapeutic resistance and tumour relapse. Thus, swift investigation of HYAL4 in more cancers could provide beneficial insight and a novel, specific treatment target for a variety of cancer patients. Together, these data suggest that a defective HYAL4 mechanism may underlie the formation of various cancers.

## Discussion

It is clear that many secrets of HYAL4 biology await discovery. A few clues buried within large data sets are beginning to emerge, demonstrating the usefulness of open access data repositories and predictive software programmes of modern science alongside traditional data publication. However, many annotations of human HYAL4 still report that the protein(s) only have hyaluronidase activity, which like the name HYAL4, is misleading and may have contributed to the slow connection of HYAL4 to CS-mediated diseases. Others have proposed new names to combat this, namely chondroitin sulphate hydrolase (CSHY; Kaneiwa et al., 2010), but unfortunately the abbreviation looks too similar to CHSY that is already used for CS synthesis enzymes. Another abbreviated name, Chase (from chondroitinase), has also been used (Lokeshwar et al., 2020) but as no sulphate reference is mentioned, it implies that HYAL4 has unsulphated chondroitin degradation activity, which is not accurate. Using “CSase” is also not advised since this abbreviation has been used historically for bacterial CS lyases. Therefore going forward, an abbreviation similar to HPSE, which is a well known GAG hydrolase, might be optimal: CHSE.

Multiple mechanisms may orchestrate the localisation and activity of human HYAL4 via expression of alternative splice variants by different cell types, although which cell types express which variants(s) remains to be investigated. The missing C-terminus of the v1 variant infers that the truncated version may be solely secreted and that the full length protein is associated with the cell surface. However, release of the putative GPI-anchor in the full length protein might also be possible via lytic cleavage or by other GPI-mitigated mechanisms (Muller, 2018). Aside from this, it is logical to assume that the full length version is primarily located on the cell surface to modulate cell surface CSPGs and the secreted version(s) are free to cleave CS in the extracellular milieu, matrices and on other cells. Close investigation of the protein structure and comparison of different variants would quickly detail which hypotheses are true, and provide leads for uncovering the mechanism(s) behind HYAL4 functions.

A limited number of useful methods and tools exist for HYAL4. The link between HYAL4 activity and expression of the mab 3B3- motif provides an effective screening tool for HYAL4 modification of CSPGs in new contexts and an anchor point to begin more specific analyses on a cell type or disease situation of interest. For example, 3B3- associated diseases such as osteoarthritis, development and aging warrant HYAL4 investigation. In addition, CSPGs (e.g., lubricin/proteoglycan 4), which display 3B3+ after cABCase digestion may also contain HYAL4 cleavage sites within the CS chains (Lord et al., 2012), meaning a wealth of knowledge may await in the literature for CSPGs modulated by HYAL4. Unfortunately, the same is not true for HYAL4 activity assays, where the lack of specific tools continues to complicate analyses due to overlapping activities for HYAL1 within CS substrates. However, as the interest in HYAL4 research grows, the production of new knockout models, antibodies, specific detection tools and assays for HYAL4 activity will enhance our knowledge of this CS degradation enzyme and will hasten its identification in organismal processes and reveal its role in cancers (and other human diseases). Understanding the role of HYAL4 in human diseases will undoubtedly provide novel diagnostic opportunities and therapeutic targets for drug discovery.

## Author Contributions

MM-H conceived the idea, wrote the review, and produced Figure 1.

## Conflict of Interest

The author declares that the research was conducted in the absence of any commercial or financial relationships that could be construed as a potential conflict of interest.

## Publisher’s Note

All claims expressed in this article are solely those of the authors and do not necessarily represent those of their affiliated organizations, or those of the publisher, the editors and the reviewers. Any product that may be evaluated in this article, or claim that may be made by its manufacturer, is not guaranteed or endorsed by the publisher.
